# Ambulance personnel use of coercion and use of safety belts in Norway

**DOI:** 10.1186/s12913-023-10332-x

**Published:** 2023-11-27

**Authors:** Kristin Häikiö, Anne Kristine Bergem, Øyvind Holst, Nina Øye Thorvaldsen

**Affiliations:** 1 OsloMet - Oslo Metropolitan University, P.O Box 4 St. Olavs Plass, N-0130 Oslo, Norway; 2 Norwegian Psychiatric Association, Sentrum, P.O Box 1152, 0107 Oslo, Norway; 3 SIEFER South-East, National Competence Network for Security, Prison, and Forensic Psychiatry, Oslo University Hospital, P.O Box 4956, Nydalen, Norway

**Keywords:** Paramedic, Emergency responders, Ambulances, Ambulance personnel, Coercion, Transportation of patients, Patient safety, Safety belts, Accidents, Occupational, Working conditions

## Abstract

**Background:**

Providing health care in a moving vehicle requires different considerations regarding safety than in other settings. Use of seatbelts are mandatory, and during ambulance transport patients are fastened to the stretcher with safety straps. However, patients who wriggle out of, or unfasten, their safety straps pose a threat to him/herself and escorting personnel in the ambulance compartment in case of an accident. To prevent harm, ambulance personnel sometimes restrain the patient or unfasten their own seatbelts to keep the patient safe on the stretcher. The prevalence of coercive measures, and the relationship between the use of mechanical restraints comparable to coercion and seatbelt use, are scarcely investigated. Use of coercion normally requires a specific statutory basis. However, coercive measures needed to ensure safety in a moving vehicle while providing healthcare is hardly discussed in the literature. The aim of this study is to explore the use of coercion in ambulance services, the use of safety belts among escorts in situations where they need to keep the patient calm during transportation, and to analyse the relationship between safety belt non-compliance and coercion in these situations.

**Methods:**

This is a retrospective, cross-sectional study using a self-administered, online survey aiming to investigate the use of coercion and use of seatbelts during ambulance transport. Approximately 3,400 ambulance personnel from all 18 Health Trusts in Norway were invited to participate between Oct 2021 and Nov 2022. Descriptive analyses were used to describe the sample and the prevalence of findings, while multiple linear regressions were used to investigate associations.

**Results:**

Altogether, 681 (20%) ambulance personnel completed the survey where 488 (72.4%) stated that they had used coercion during the last six months and 375 (55.7%) had experienced ambulance personnel or escorting personnel working with unfastened seatbelts during transport. The majority of respondents experienced coercion as being unpleasant and more negative feelings were associated with less use of seatbelts.

**Conclusions:**

Coercion seems to be used by ambulance personnel frequently. For the study participants, keeping the patient securely fastened was prioritized above escorting personnel’s traffic safety, despite feeling uncomfortable doing so. Because coercive measures have negative consequences for patients, is associated with negative feelings for health personnel, and is not discussed ethically and legally in relation to the prehospital context, there is an urgent need for more research on the topic, and for legal preparatory work to address the unique perspectives of the prehospital context in which traffic safety also is an important factor.

**Supplementary Information:**

The online version contains supplementary material available at 10.1186/s12913-023-10332-x.

## Background

The use of coercion may be inconsistent with human rights-based mental healthcare. In a literature review aiming to examine the extent and nature of coercive practices in mental healthcare, coercion is described as “a global challenge that requires urgent action” [1]. The World Health Organization’s (WHO’s) European Mental Health Action Plan 2023–2020 ([2] p. 5) states that “all steps should be taken to promote voluntary admission and treatment, and avoid coercion.”. In the Norwegian constitution, as in many other countries, the principle of legality suggests that the state cannot intervene in citizens’ lives unless a specific statutory basis is present [3]. In Norway, the use of restraints in mental health services is described as one of the priority areas in the Norwegian patient safety program “In safe hands 24–7” [4] In a prehospital context, however, the mandatory use of seat belts in a moving vehicle adds another perspective which is not discussed in the coercion literature.

In Norway, the use of safety belts in vehicles has been compulsory since 1988 [5, 6]. Patients who are agitated or incapable or unwilling to cooperate with ambulance personnel can be difficult to secure properly during transport. This is partly because they can easily wriggle out of the stretcher’s safety straps and partly because they can easily access the buckle to unfasten the straps. In such situations, ambulance personnel must act according to their best judgement and discretion to ensure their own safety and that of the patient [7]. Research has shown that ambulance personnel frequently encounter patients who are confused, agitated, and sometimes violent, and coercive measures are used to protect both the patient and the ambulance personnel from harm [8, 9]. The use of coercion is frequently debated in the mental health literature, and numerous research reports discuss the use of coercion towards mental health inpatients [10, 11] and the lack of therapeutic value from coercion [1, 12]. In the ambulance context, however, the literature on coercion is scarce.

The prevalence of coercion in ambulance settings is rarely reported [8, 13, 14]. In this study we report on the use of coercion on patients related to the use of safety belts for escorting personnel because research indicates that ambulance personnel often unfasten their own safety belts when providing treatment to a patient in the ambulance compartment [14–16]. In this paper we illuminate that in a prehospital context the use of coercion must be discussed in relation to a safe ambulance transport which encompass a broader perspective than the debate on coercion in mental health institutions because of the traffic safety aspect.

Coercion is not described in a unified manner in either research [17] or in health legislation, but “overcoming resistance” is one definition that is recommended in Norwegian policy ([18] p. 365). In this study, we juxtapose coercion with the use of physical force, which includes restraining or holding a patient’s arms or legs. The latter is a type of coercion that, according to previous research, is commonly used among Norwegian ambulance personnel [13], sometimes without the ambulance personnel being aware that it is a coercive measure [14, 19].

The aim of this study is to explore the use of coercion in Norwegian ambulance services; the use of safety belts among escorting personnel in the ambulance compartment during transports, related to keeping the patients calm; and the relationship between the use of coercion and the ambulance personnel’s use of safety belts.

## Methods

### The setting of the study

Most hospital care in Norway is provided through the country’s 20 public hospital trusts, which are state-owned and governed as publicly-owned corporations, often referred to as the four regional health trusts [20]. The ambulance services are departments under 18 of the hospital trusts. Ambulance services are, like public hospitals, tax-financed and free of charge. According to Statistics Norway, there were 5,232 ambulance personnel in Norway in 2022 and approximately 3,400 (65%) of those were active personnel in the ambulance services [21, 22].

*Ambulance* personnel is the term used in this paper to describe those employed by ambulance services to work in ambulances, irrespective of their level of education. Their education level varies from apprentices who have completed two years of upper secondary school training only, emergency medical technicians (EMTs) who have completed two years of upper secondary school training and two additional years of apprenticeship in the ambulance service, paramedics who are EMTs with additional paramedic education at university/university college level (usually 1 year), and paramedicines who have completed a bachelor degree in paramedicine. In addition, around 20% of those working as ambulance personnel are nurses, with a bachelor’s degree in nursing. Nurses and paramedicines may also have a master’s degree or higher university education. Occasionally, other types of healthcare personnel work in ambulance services, usually as substitutes. The term *escort* includes other professionals, such as police officers, assisting the ambulance personnel.

### Study design and recruiting participants

Based on the findings in one of the authors previous works [14, 19], and her own experience from working in ambulance services, the authors developed a self-administered online survey aiming to investigate the use of coercion and the use of safety belts by escorts nationally. The questions about the use of coercion and safety belts were previously piloted in Innlandet health trust, and the results are published elsewhere [13].

An email with an information letter about the study and a link to the survey was sent to the managers of the prehospital/ambulance departments of all 18 hospital trusts in Norway in October 2021. A web-based survey tool developed by the University of Oslo, called “Nettskjema”, was used with an open link to facilitate easy access to the survey, make it possible for participants to forward the link to other potential responders, and at the same time avoiding the collection of personal data. No other incentives were offered to the participants. The managers were asked to share information about our study with their employees and encourage them to volunteer their participation. The invitation to participate, along with information about the study, was also shared in relevant groups on Facebook and advertised through the Ambulanseforum website. Due to lack of response, the author NØT phoned the health trust managers in February 2022. They all agreed to share the survey with their employees by email and/or on their local information platform. Information and a link to the survey was also shared on NAKOS’ (National competence service for prehospital emergency medicine) website [23]. To start the survey, the participants needed to actively tick a box stating that they had read the information letter, were participating voluntarily, and that they consented to the information they provided being used in the project.

### The variables

**Fig. 1:**
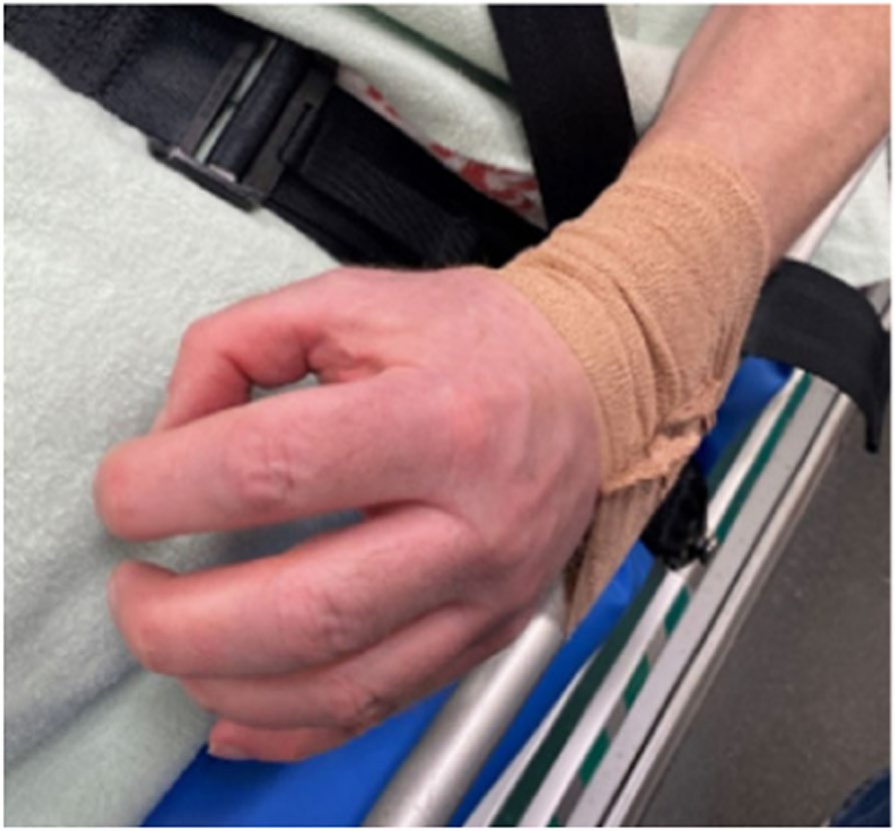
Illustrative picture of how arms can be restrained with self-adherent bandage. Photo: Tiril Thorvaldsen Fosen

Our data cover the following variables, which were coded as follows:

- Number of callouts where respondents used coercion (discrete variable 0 to > 10). The variable is the answer to the question: “In the last six months, try to indicate on how many callouts you needed to use coercion to ensure that the patient was safely secured during transport”.
- Number of callouts where respondents restrained patients’ arms or legs (discrete variable 0 to > 10). The variable is the answer to the question: “In the last six months, try to indicate on how many callouts you restrained the patients’ arms and/or legs using blankets/bandages/velcro straps etc. to ensure that the patient was safely secured during transport”. Example is shown in Fig. 1.
- Number of callouts where escorts were not seated with their safety belts fastened (discrete variable 0 to > 10). The variable is the answer to the question: “In the last six months, try to indicate on how many callouts you experienced that ambulance personnel/police/others sitting without seatbelts on to keep a patient calm during transport”.

Participant age. To ensure anonymity for the respondents, the “years of age” variable was collected as age groups. See Supplementary file for details.

Participant gender, male/female.

Education level, coded as lower/upper secondary school, usually students in an apprenticeship; emergency medical technician; up to two years of university level education; up to four years of university level education; four years or more of university education.

Years of experience from ambulance services was collected in groups of years to ensure anonymity.

Working hours is measured as “close to full-time position” = 25,5–33,5 h per week; “51–75% position” = 17–25 h per week; “26–50% position” = 9–17 h per week; “ < 26% position” = < 17 h per week.

Experience with use of coercion. Participants were asked how they experienced having to use coercion to safely secure a patient during ambulance transport. They answered on a scale of 0–10, indicationgfrom completely unproblematic to very uncomfortable.

To see the full survey in the original language and English, see Supplementary files.

### Analyses

Descriptive analyses and multiple linear regression analyses were conducted by the first author, who has previous experience with regression analysis, in the software IBM SPSS statistics version 28 [24]. Analysis was discussed with a more experienced statistician. For discrete counted variables with normal distribution, the results are given as mean and standard deviation (sd). Discrete, counted variables that are not normally distributed are given as median, and minimum and maximum value. Nominal variables are given with number of cases (n), per cent (%), and missing values. The threshold *p*-value was considered statistically significant when < 0.05.

## Results

We received 681 completed replies representing all 18 health trusts in Norway, as shown in Table 1. This indicates a response rate of approximately 20%.

**Table 1:** Distribution of respondents between different regions, *N* = 681, missing = 0

**Ambulance district**		
**SOUTH-EASTERN NORWAY REGIONAL HEALTH TRUST**	**450**	**(66.1)**
Innlandet hospital trust, n (%)	164	(24.1)
Oslo university hospital trust, n (%)	101	(14.8)
Østfold hospital trust, n (%)	50	(7.3)
Hospital of Southern Norway, n (%)	44	(6.5)
Telemark hospital trust, n (%)	22	(3.2)
Vestfold hospital trust, n (%)	35	(5.1)
Vestre Viken hospital trust, n (%)	34	(5.0)
**CENTRAL NORWAY REGIONAL HEALTH TRUST**	**69**	**(10.1)**
St. Olav hospital trust, n (%)	48	(7.0)
Nord-Trøndelag hospital trust, n (%)	21	(3.1)
**WESTERN NORWAY REGIONAL HEALTH TRUST**	**100**	**(14.7)**
Fonna hospital trust, n (%)	29	(4.3)
Bergen hospital trust, n (%)	21	(3.1)
Møre og Romsdal hospital trust, n (%)	19	(2.8)
Førde hospital trust, n (%)	25	(3.7)
Stavanger hospital trust, n (%)	6	(0.9)
**NORTHERN NORWAY REGIONAL HEALTH TRUST**	**62**	**(9.1)**
Nordland hospital trust, n (%)	25	(3.7)
Northern Norway health authority, n (%)	19	(2.8)
Finnmark hospital trust, n (%)	12	(1.8)
Helgeland hospital trust, n (%)	6	(0.9)

### Description of the sample

The age group 35–39 years represents the central tendency of the age group of the respondents. Slightly more than one third (*n* = 255, 37.4%) were females and the median years of experience from working in the ambulance services was 12 years (range 0–22 years). The majority (81.8%) of respondents worked full-time (25–33.5 h per week) in an emergency ambulance service. The remaining participants worked part-time with less than 25 h per week. See Table 2.

**Table 2:** Characteristics of the sample, *N* = 681

Participant characteristics			Missing
Female, n (%)	255	(37.4)	0
Age group in years, median (min–max)	35–39	(< 19—> 65)	0
Years of experience, median (min–max)	12	(0–22)	7
Working close to full-time, n (%)	557	(81.8)	0

With respect to education, the largest group was licenced paramedics with no university education (*n* = 276, 40.5%). The second largest group was licenced paramedics with up to two years of university education (*n* = 172, 25.3%). Another large group comprised paramedics with 3–4 years of university education (*n* = 141, 20.7%). The remaining 13.5% of participants had lower/upper secondary school (n = 40, 5.9%) or had more than four years of university education (*n* = 52, 7.6%).

Most respondents had close to a full-time position or full-time position (*n* = 559, 82.1%). Among the remaining participants, 44 (6.5%) worked 17–25 h per week, 45 (6.6%) participants worked 9–17 h per week, and 33 (5.8%) worked less than 17 h per week.

### Frequent use of coercion among ambulance personnel

During the last 6 months, 488 (72.4%) of the 674 (*N* = 681, 7 missing) ambulance personnel stated that they had used coercion. Among these, 349 (71.5%) respondents had used coercion 1–3 times, while 186 (27.6%) respondents reported no use of coercion during the last 6 months. See Table 4.

When asked about fastening patients’ extremities with blankets/bandages/velco straps or similar equipment, 243 (36.1%) respondents confirmed to have used such measures during the last six months. See Table 2.

Of the 173 respondents who stated that they had not used coercion within the last six months, 13 (7.5%) of them reported having restrained patients’ extremities, and 30 (4.5%) reported more frequent use of restraining patients’ extremities with straps or similar equipment than they reported the use of coercion, as shown in Table 3.

**Table 3:** Participants reporting on the use of coercion vs restraining patients’ arms or legs

		Number of callouts where respondents restrained patients’ arms or legs									Number of callouts without reported coercion but with restrained arms or legs
		**0**	**1**	**2**	**3**	**4**	**5**	**6**	**9**	** > 10**	
Number of callouts where respondents used coercion during transport	**0**	173	7*	1*	0	1*	3*	0	0	1*	**13**
	**1**	93	30	2*	2*	1*	1*	0	0	1*	**7**
	**2**	82	16	26	2*	1*	0	0	0	0	**3**
	**3**	40	23	17	8	0	3*	1*	0	0	**4**
	**4**	20	10	12	7	4	1*	1*	0	0	**2**
	**5**	10	4	8	8	2	5	0	0	0	**0**
	**6**	2	1	4	0	2	0	1	0	1	**0**
	**7**	2	0	2	0	1	0	0	0	0	**0**
	**8**	6	0	1	2	0	0	1	0	0	**0**
	** > 10**	3	0	0	4	3	4	1	2	4*	**1**
***Total***		**431**	**91**	**73**	**33**	**15**	**17**	**5**	**2**	**7**	**30**

*respondents have reported more frequent use of restraining patients’ extremities with straps or similar equipment than they reported the use of coercion

### Frequently working in the ambulance compartment without being seated with safety belts fastened

In response to the question of how many times they had experienced escorts (ambulance personnel, police, or other escorts) not being seated with their safety belt fastened during transport due to the need to hold or secure the patient, 375 (55.6%) respondents confirmed they had experienced this in the last 6 months. See Table 4.

**Table 4:** Frequency of callouts within the last six months, where respondents restrained patients’ arms og legs, used coercion, or witnessed that escorts were not seated with their seat belts on (*N* = 674, missing = 7)

	Number of respondents who used coercion		Number of respondents who restrained patients’ arms or legs		Number of respondents who witnessed that escorts were not seated with their seat belts on^a^	
	**n**	**(%)**	**n**	**%**	**n**	**%**
**On 0 callouts**	186	(27.6)	431	(63.9)	299	(44.4)
**On 1 – 3 callouts**	349	(51.8)	197	(29.2)	263	(39.0)
**On 4 – 6 callouts**	103	(15.3)	37	(5.5)	84	(12.5)
**On > 6 callouts**	36	(5.3)	9	(1.3)	28	(4.2)

^a^note the description of the variable in the Method section

### Using coercion was experienced as problematic

On a scale of 0–10 (0 = unproblematic, 10 = very unpleasant), the respondents reported a mean score of 7 (sd 2.3, min–max 0–10) describing how problematic they experienced callouts where coercion was used.

### Prediction of not being safely seated

More problematic experiences with using coercion and more frequent use of coercion were associated with escorts more frequently not being seated with their safety belts fastened, as shown in Table 5.

**Table 5:** Multiple linear regression analysis predicting escorts being seated without safety belt fastened during transport (*N* = 874, missing = 7)

R^2^ 0.203	Unstandardised coefficients		Standardized Coefficients	Sig	95.0% confidence interval for B		Collinearity statistics	
	B	Std. Error	Beta		Lower Bound	Upper Bound	Tolerance	VIF
(Constant)	.181	.352		.607	-.509	.871		
Years of experience	-.006	.012	-.018	.630	-.029	.017	.889	1.125
Level of education	-.043	.074	-.020	.560	-.188	.102	.978	1.022
Participant gender, male	.161	.172	.035	.349	-.176	.498	.866	1.155
Participant experience with use of coercion	.092	.034	.096	.007*	.025	.158	.950	1.053
Number of callouts where respondents used coercion	.433	.034	.440	< .001*	.366	.499	.994	1.006

### Predictions of using coercion

There was no significant association between the number of years of experience, level of education, gender, age, or experience of using coercion with more frequent restraining of patients’ extremities. This is reflected in the results from the multiple linear regression model shown in Table 6.

**Table 6:** Multiple linear regression analysis predicting the restraining of patients' extremities during ambulance transport (*N* = 874, missing = 7)

R^2^ 0.002	Unstandardised coefficient		Standardized coefficients		95.0 confidence interval for B		Collinearity statistics	
	B	Std. Error	Beta	Sig	Lower Bound	Upper Bound	Tolerance	VIF
Constant	.799	.299	.033	.008	.211	1.387		
Years of experience	.008	.014	.000	.590	-.020	.035	.407	2.457
Level of education	.000	.061	.028	.996	-.120	.120	.963	1.039
Participant gender	.095	.142	-.093	.502	-.183	.374	.855	1.170
Participant age	-.070	.046	.064	.129	-.161	.021	.398	2.514
Participant experience with use of coercion	.045	.028	.033	.104	-.009	.100	.947	1.055

## Discussion

A national, retrospective, online survey distributed to ambulance personnel in Norway resulted in 681 completed online questionnaires showing that ambulance personnel commonly used coercion. Securing the patients’ extremities was one type of coercion that was used, although restraining of patients’ arms and/or legs were not always acknowledged as coercion. The results also indicate that many ambulance personnel were uncomfortable with using coercion to keep the patient calm, and feeling more uncomfortable was a predictor for ambulance personnel not being safely seated with their safety belts fastened during transport.

To our knowledge, the prevalence of coercion in the ambulance services is scarcely covered in previous research. However, the high prevalence (74.2%) in this study is not surprising. A study from Poland found that 75% of emergency personnel at some point had immobilised patients [8]. Researchers have found that emergency medical service providers, such as ambulance personnel, have frequent encounters with patients that are agitative or violent [8, 25]. Such encounters can result in significant injury to the patient and/or to escorts, especially during ambulance transport. When the ambulance is moving, there is nowhere to escape from the situation, and it is not always possible or safe to stop the vehicle, e.g. when driving on busy, high-speed roads. Coercion is used among health personnel to prevent injuries to the patient and personnel and to facilitate the provision of necessary medical care to people who resist help due to acute illness or confusion, but lacks the capacity to consent [25]. For the ambulance personnel, the matter of traffic safety for both the patient and escorting personnel (everyone in the ambulance compartment) must also be considered. The latter is unique to ambulance personnel work environment and poses a perspective that is not commonly discussed The lack of debate on the prehospital perspectives leaves ambulance personnel in situations where they lack support for their everyday difficult ethical and legal decisions.

Our model for predicting the restraint of patients’ arms and legs showed no significant associations with ambulance personnel age, education, years of experience, gender, or experience of using coercion. In fact, years of experience, level of education, gender, age, and experience of using coercion only explained 0.2% of the proportion of variance in restraining patients’ arms and legs. This indicates that other variables are more predictive for these actions. More research is therefore needed to explore these variables and discuss whether these ad-hoc solutions to prevent patients from unfastening the straps on their stretchers are ethically and legally acceptable in this context.

**Fig. 2:**
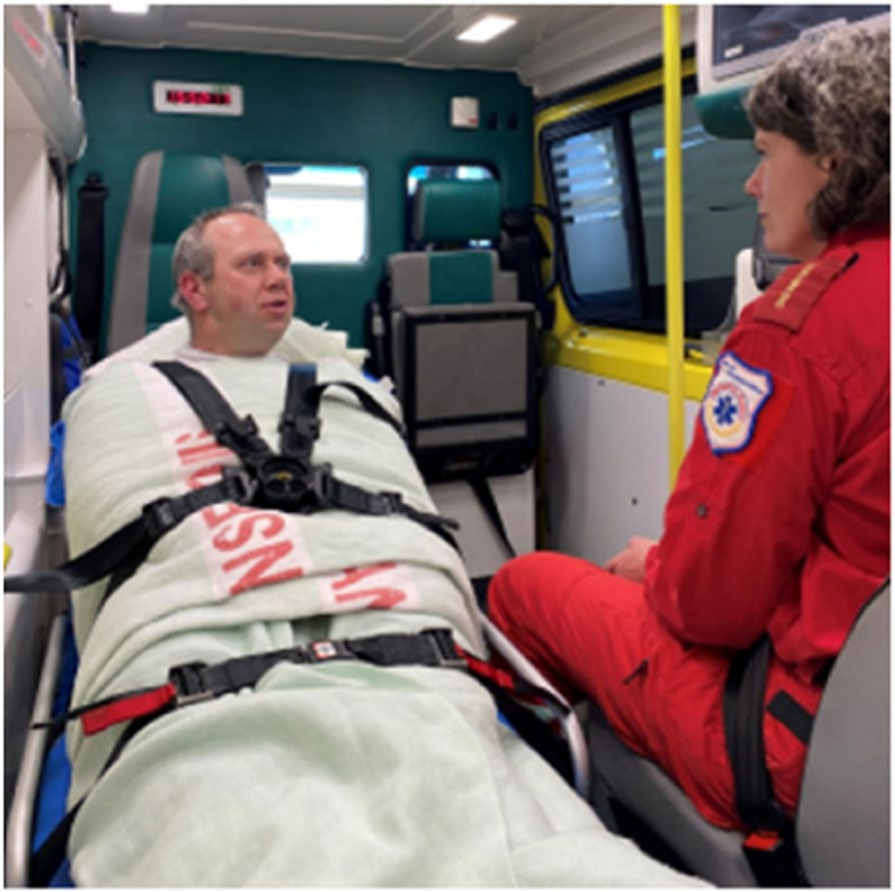
Illustrative picture of a person being restrained on the ambulance stretcher. Photo: Tiril Thorvaldsen Fosen

As mentioned, there is no unambiguous definition of coercion, and the results show that ambulance personnel do not always recognise the restriction of patients’ extremities as coercion. In Fig. 2, we show an example of how patients can be restrained during transport in a way that prevents confused, agitated, or violent patients from unfastening their safety belt, tinkering with ambulance equipment, or from accidentally or purposely hitting ambulance personnel. This is in line with findings described in a qualitative study exploring use of coercion in the Norwegian ambulance services [14, 19]. Restricting the patients’ extremities may prevent incidents that require ambulance personnel to unfasten their own safety belt, but using mechanic coercion (such as restraining patient’s arms and legs) often feels uncomfortable and a study among Norwegian nurses in a mental health ward reports that using of mechanical restraints were often chosen as the last resort [26]. Mechanical restraints are forbidden for ethical reasons in countries such as UK and Iceland [27], and the perceived ethical burden of using mechanical restraints combined with the lack of appropriate equipment for mechanical restraints in the Norwegian ambulances, may be some of the explanation for why ambulance escorts choose to unbuckle their own safety belts to calm the patient down by holding them rather than restraining them. For practical reasons, such as the placing of the seats in the ambulance compartment, it is hardly possible for the escorts to be safely seated with seatbelts on and at the same time hold the patient down and safely secured to the stretcher. Keep in mind that patients are able to open the buckle on the safety straps or try to wriggle out of them [27].

However, the restraint of a patient’s arms and legs must be considered coercion according to the definition used in this study if the restraint is used to overcome the patient’s resistance of healthcare. At the same time, while the use of safety belts in vehicles is mandatory for everyone under Norwegian legislation, ambulance personnel are obliged to provide the necessary medical care to patients who may not have the capacity to consent and may or may not be agitative, confused, or violent. This dilemma illustrates a common situation for ambulance personnel, yet it is an area that is not addressed in either health legislation or traffic legislation. In addition, the ethical and legal grounds for using coercion on patients with mental health issues outside of the mental health services is unclear and debated [28, 29]. This unclear legal ground makes ambulance personnel insecure about their legal rights to use coercion to facilitate a safe transport [14]. Medical care and transport provided by ambulance personnel in an ambulance is considered healthcare, but the prehospital setting has never been extensively addressed in legal preparatory works [13]. The use of coercion in ambulance services in relation to traffic safety and the patient’s right to necessary medical help is barely covered in the research literature, except in a recent published paper from Norway [14].

Our results have shown that Norwegian ambulance personnel frequently work in the ambulance compartment without being safely seated with their safety belts fastened, and the regression analysis indicates an association between the need to use coercion to keep the patient calm and the escorting personnel not wearing their safety belts. Our regression model explained 20% of the variances (R square = 0.2). For social sciences this is an acceptable R square on the condition that some of the predictors are statistically significant and that there is no multi-collinearity among the explanatory variables [30]. Our model meets these requirements. In addition, we are not trying to predict human behaviour, but rather the goal is to assess whether specific predictors have a significant effect on the dependent variable, which is also a reason to accept the often low R square value in social sciences [30].

In a Norwegian interview study, Thorvaldsen [19] found that one reason ambulance personnel unfastened their seatbelt was to keep the patient from removing their safety straps and getting up from the stretcher during transport. Findings from many countries, including Finland, UK, USA, and Thailand, show that ambulance personnel regularly unfasten their safety belts to work in the ambulance compartment [31–33]. One research study reported that ambulance personnel safety belt use was lower than normal when the patient was critically ill [31].

Knowing that not using a safety belt increases the risk of dying in a traffic accident by 60% [6], the safety of ambulance personnel in the ambulance compartment during transport decreases significantly when they unfasten their belts. Our results and prior research results indicate that when the patient needs extensive attention or intervention during transport, this poses a risk for ambulance personnel who then unfasten their own belts to ensure the patient’s safety.

There seems to be a trade-off between the wish to reduce coercion on patients and the traffic requirement for a safe transport for patients who are not cooperating but are in need for medical help. A legal and ethical debate about the use of coercion in ambulance settings is therefore necessary along with more research on the topic, in order to improve the quality of ambulance services for patients and ensure the safety of patients and ambulance personnel alike.

Training ambulance personnel to raise their awareness of what coercion is would appear appropriate but will not solve the problem entirely as long as the legal preparatory work has not addressed the unique perspectives of the prehospital context.

Our participants reported a mean score of seven (from 0 to 10) when asked how problematic they experienced situations where they had to use coercion. This high reported mean score indicates that ambulance personnel find the use of coercion burdensome and stressful, even though it might be necessary. This is in line with findings from a systematic literature review reporting from studies which investigated nursing staff in acute mental health services [12]. Our regression analysis indicates an association between reporting high emotional burden when using coercion and personnel not being seated with their safety belts on. One possible explanation for this association could be that when ambulance personnel find it more problematic to use coercion, they are less likely to use mechanical restraints. Although holding a patient’s hands or arms may also be a way of overcoming their resistance, it may be viewed by some as more caring and less intrusive. Consequently, ambulance personnel or other escorts in the ambulance compartment (e.g. the police) might be more likely to unfastened their own seatbelt to ensure the patient’s safety and provide caring treatment at the cost of their own personal safety. A study from Poland found that having more than five years of work experience was associated with being less likely to use coercion with the patient than was the case with less experience [8]. We did not find a significant association between age and the use of coercion, nor between experience and use of coercion in our models, however.

Another possible explanation for the unpleasant experience of using coercion might be, as we have reported in a previous paper, the lack of legal preparatory work to address the unique perspectives of the prehospital context [13]. The legal basis for using coercion outside the scope of the Mental Health Act is unclear and in Norway it is debated who are allowed to use coercion outside of mental health institutions and how the legislations should be interpreted prehospitally [34]. It is understandable that ambulance personnel are insecure about their rights to use coercion, especially since ambulances are not equipped to restrain patients except with their safety belts. The challenge is that all patients are able to open the safety belt bucked or wriggle out of their safety belts if they choose to – unless they are restrained to the stretcher with other measures, such as shown in Figs. 1 and 2. As the pictures in this paper show, ambulance personnel use ad-hoc solutions to secure the patient in the ambulance compartment, meaning the ambulances are not sufficiently equipped to handle agitated or confused patients in a way that is based on consensus interpretation of the legislations.

The strengths of this study include the high number of participants, participation from all 18 hospital trusts in Norway and participation from urban and rural areas in different geographical regions of Norway. The distribution of participants age and gender were similar to that of the population of ambulance personnel in Norway and both rural and urban areas were represented. The proportion of participants working close to full-time is approximately the same in our study (81.8%) as the national average for ambulance personnel, where 91% work full-time ([29] p.157).

Due to the low response rate and the limited national statistics on the characteristics of ambulance personnel it is not possible to sufficiently assess the generalizability of the sample. Based on the number of participants from each regional health trust, we assess that the South-East Regional Health Trust is overrepresented and that the Northern Regional health trust is underrepresented, which indicate a response bias [35], meaning that responders does not represent the study population of Norwegian ambulance personnel.

The open link to the survey makes it possible for participants to reply more than once. However, we consider this risk to be low and consequently a low risk of having significantly changed the results.

Limitations in this study are typical of self-reporting, retrospective studies, that there might be a significant risk of recall bias such as inaccurately reporting [35, 36]. In addition, there is a low response rate, and participants may be those who find this subject particularly interesting, while others are not represented. Consequently, readers should critically evaluate the method and carefully interpret the results.

Because the questions used in this study were not tested or validated for the purpose, we do not know if the questions are understood consistently across participants or across time. This represents a potential measurement error and can cause “noise” into measures of variables and into the relationship between variables [37]. This potential measurement error and the risk of recall bias and response bias, calls for caution when interpreting the results and generalising to other populations.

Participation in this study is based on voluntary, informed consent, and the participants are anonymous. The data were stored according to the current regulations and local procedures at OsloMet University. As mentioned above, the legal basis for using coercion in ambulance services is debated and the prehospital environment was not discussed in the legal preparatory work. For this reason, participants may have reported actions that must be considered to be in the grey zone of legislations or even illegal. Hence, an anonymous study was important to reduce bias.

## Conclusion

Coercion seems to be used regularly by ambulance personnel in Norway to ensure safe patient transports. Although the majority of our study participants felt uncomfortable using coercion, they often used coercive measures to ensure the patient was securely fastened during transports, and this was prioritized above escorting personnel’s traffic safety. Coercive measures have negative consequences for patients and feels uncomfortable for escorts, but are still used frequently. There is a need to discuss how coercion can be reduced in ambulance services. When coercion is the only viable option for ensuring a safe transport we need to discuss how this can be carried out in a caring and sensitive way that does not compromise the safety of the patient or the escorting personnel. A legal and ethical debate about the use of coercion in ambulance settings is necessary, along with more research on the topic. Researchers, policy makers, and clinicians must work together to find solutions to promote good quality ambulance care for patients and a safe work environment for escorts.

### Supplementary Information

**Additional file 1.**

**Additional file 2.**

**Additional file 3.**

## Data Availability

The datasets used and/or analysed during the current study are available from the corresponding author on reasonable request.

## Abbreviation

NØT: Nina Øye Thorvaldsen (last author)
