# Supplementary material for: Ambulance personnel use of coercion and use of safety belts in Norway
Source: BMC Health Serv Res. 2023 Nov 27;23:1303. doi: 10.1186/s12913-023-10332-x (PMC10680207; doi:10.1186/s12913-023-10332-x)
Supplement: Supplementary file 1 — Additional file 1. [file 12913_2023_10332_MOESM1_ESM.docx]

Use of Force in Securing Patients during Ambulance Transport

The purpose of this survey is to gather knowledge about how often physical force is used to secure patients during ambulance transport. Here, the use of force is equated with the use of physical power.

Link to the information sheet about the survey: [Link]

I hereby consent to participate in the survey "Use of Force in Securing Patients during Ambulance Transport". I have read the information about the survey and hereby consent that the information I provide here can be used in the project.

Yes, I consent

First, we would like some background information about you.

Please specify your gender:

Male Female

# Please specify your age:

<19 years
20-24 years
25-29 years
30-34 years
35-39 years
40-44 years
45-49 years
50-54 years
55-59 years
60-64 years
< 65 years

# Please specify your highest completed education?

Lower/upper secondary school (3-year)
Emergency medical technician
Up to 2 years College or university education
Up to 4 years College or university education
More than 4 years of College or university education

# Which health trust are you employed in?

If you are employed in several trusts; tick the trust in which you have the highest percentage position or work the most.

Finnmark hospital trust
Nordland hospital trust
Helgeland Hospital trust
North-Trøndelag hospital trust
St. Olavs Hospital trust
Møre og Romsdal hospital trust
Bergen hospital trust
Førde hospital trust
Fonna hospital trust
Stavanger hospital trust
Innlandet hospital trust
Oslo University hospital trust
Vestre Viken hospital trust
Vestfold hospital trust
Telemark hospital trust
Østfold hospital trust
Hospital of Southern Norway

# Do you work in operational service on an ambulance performing emergency and transport assignments?

Yes No

The survey is aimed at ambulance personnel working operationally. By operational, we mean ambulance personnel working on ambulances performing emergency and transport assignments. If you answered no to the previous question but work operationally, please try again. If you do not work operationally, we thank you for being willing to answer the survey.

**Indicate your percentage of operational service in the last six months?**

*This item is only displayed if the option "Yes" is selected in the question "Do you work in operational service on an ambulance performing emergency and transport assignments?"*

< 25%
26-50%
51-75%
>75%

# How many years of experience do you have from operational service on an ambulance performing emergency and transport assignments?

*This item is only displayed if the option "Yes" is selected in the question "Do you work in operational service on an ambulance performing emergency and transport assignments?"*

Example: if you have worked in ambulance service since 2018, you have approximately three years of experience (regardless of whether you are a temporary or permanent employee)

< 1 year of experience
1-2 years of experience
2-4 years of experience
5-6 years of experience
7-8 years of experience
9-10 years of experience
11-12 years of experience
13-14 years of experience
15-16 years of experience
17-18 years of experience
19-20 years of experience
< 20 years of experience

***In the next three questions, we ask you to think back over the last six months.***

# In the last six months, try to indicate on how many callouts you needed to use coercion to ensure a patient was safely secured during transport?

*This item is only displayed if the option "Yes" is selected in the question "Do you work in operational service on an ambulance performing emergency and transport assignments?"*

For example, by: putting on the patient's seat belts if the patient tries to get out of them, holding the patient's arms and/or legs without the patient's consent

0
1
2
3
4
5
6
7
8
9
10 or more

# In the last six months, try to indicate on how many callouts restrained the patient's arms and/or legs using blankets/bandages/Velcro straps etc. to ensure that the patient was safely secured during transit?

*This item is only displayed if the option "Yes" is selected in the question "Do you work in operational service on an ambulance performing emergency and transport assignments?"*

0
1
2
3
4
5
6
7
8
9
10 or more

# In the last six months, try to indicate on how many callouts you have experienced ambulance personnel/police/others sitting without seat belts to keep a patient calm during transit?

*This item is only displayed if the option "Yes" is selected in the question "Do you work in operational service on an ambulance performing emergency and transport assignments?"*

0
1
2
3
4
5
6
7
8
9
10 or more

**The following questions are NOT limited to a specific time period.**

# Have you ever, during your career, fastened/secured a patient's arms and/or legs using blankets/bandages etc. to ensure they were safely secured during ambulance transport?

*This item is only displayed if the option "Yes" is selected in the question "Do you work in operational service on an ambulance performing emergency and transport assignments?"*

Yes No Don't know

# Have you ever, during your career, used force in ways other than described in previous questions to secure patients during ambulance transport?

*This item is only displayed if the option "Yes" is selected in the question "Do you work in operational service on an ambulance performing emergency and transport assignments?"*

If yes, please describe in your own words [open text field] (Please do not provide information that can identify you or other individuals, either directly or indirectly)

How do you feel when force has to be used to secure a patient during ambulance transport?

*This item is only displayed if the option "Yes" is selected in the question "Do you work in operational service on an ambulance performing emergency and transport assignments?"*

Indicate your experience of such transports from completely unproblematic to very uncomfortable on the linear scale.[picture of numeric scale 0-10]

Can you say something about how you experience transports of patients where force has to be used to secure the patient during the transport?

*This item is only displayed if the option "Yes" is selected in the question "Do you work in operational service on an ambulance performing emergency and transport assignments?"*

Please describe in your own words [open text field] (Please do not provide information that can identify you or other individuals, either directly or indirectly)
