# Supplementary material for: Ambulance personnel use of coercion and use of safety belts in Norway
Source: BMC Health Serv Res. 2023 Nov 27;23:1303. doi: 10.1186/s12913-023-10332-x (PMC10680207; doi:10.1186/s12913-023-10332-x)
Supplement: Supplementary file 2 — Additional file 2. [file 12913_2023_10332_MOESM2_ESM.docx]

Bruk av tvang ved sikring av pasienter under ambulansetransport

Formålet med denne undersøkelsen er å skaffe kunnskap om hvor ofte fysisk tvang benyttes for å sikre pasienter under ambulansetransport. Bruk av tvang er her sidestilt med bruk av fysisk makt.

Lenke til informasjonsskrivet om undersøkelsen: https://docs.google.com/document/d/1yTPP4LOj57o1Xut9hAEjBUngondoPt50X8fP6jwsFlw/edit?usp=s

# Jeg samtykker med dette til å delta i spørreundersøkelsen "Bruk av tvang ved sikring av pasienter under ambulansetransport".

Jeg har lest informasjonen om undersøkelsen og samtykker med dette til at opplysningene jeg gir her kan brukes i prosjektet.

Ja, jeg samtykker

# Først ønsker vi noen bakgrunnsopplysninger om deg. Vennligst oppgi kjønn

Mann Kvinne

# Vennligst oppgi alder

>19 år
20-24 år
25-29 år
30-34 år
35-39 år
40-44 år
45-49 år
50-54 år
55-59 år
60-64 år
< 65 år

# Vennligst oppgi høyeste fullførte utdannelse?

Videregående skole, gymnas eller yrkesskole (3-årig)
Fagbrev
Høyskole eller universitetsutdanning, til og med 2 år
Høyskole eller universitetsutdanning, til og med 4 år
Høyskole eller universitetsutdanning, mer enn 4 år

# Hvilket helseforetak er du ansatt i?

Hvis du er ansatt i flere foretak; kryss av for det foretaket du har høyest prosentstilling i eller jobber mest i.

Helse Finnmark
Universitetssykehuset Nord-Norge
Nordlandssykehuset
Helgelandssykehuset
Helse Nord-Trøndelag
St. Olavs Hospital
Helse Møre og Romsdal
Helse Bergen
Helse Førde
Helse Fonna
Helse Stavanger
Sykehuset Innlandet
Oslo universitetssykehus
Vestre Viken
Sykehuset i Vestfold
Sykehuset i Telemark
Sykehuset i Østfold
Sørlandet sykehus

# Jobber du i operativ tjeneste på ambulanse som utfører akutt- og transportoppdrag?

Ja Nei

Undersøkelsen er rettet mot ambulansepersonell som jobber operativt. Med operativt mener vi ambulanspersonell som jobber på ambulanse som utfører akutt- og transportoppdrag. Svarte du nei på forrige spørsmål, men jobber operativt? Prøv igjen. Hvis du ikke jobber operativt takker vi for at var villig til å svare på undersøkelsen.

# Angi stillingsprosenten i operativ tjeneste de siste seks månedene?

*Dette elementet vises kun dersom alternativet «Ja» er valgt i spørsmålet «Jobber du i operativ tjeneste på ambulanse som utfører akutt- og transportoppdrag?»*

Med operativ tjeneste mener vi en stilling på ambulanse som ufører akutt- og transport oppdrag.

< 25%
26-50
51-75%
> 75%

# Hvor mange års erfaring har du fra operativ tjeneste på ambulanse som utfører akutt- og transportoppdrag?

*Dette elementet vises kun dersom alternativet «Ja» er valgt i spørsmålet «Jobber du i operativ tjeneste på ambulanse som utfører akutt- og transportoppdrag?»*

Eksempel: har du jobbet i ambulansetjeneste siden 2018, så har du ca tre års erfaring (uavhengig om du er vikar eller fast ansatt)

< 1 års erfaring
1- 2 års erfaring
2-4 års erfaring
5-6 års erfaring
7-8 års erfaring
9-10 års erfaring
11-12 års erfaring
13-14 års erfaring
15-16 års erfaring
17-18 års erfaring
19-20 års erfaring
< 20 års erfaring

**I de tre neste spørsmålene ber vi deg tenke tilbake på de siste seks månedene**

I løpet av de siste seks månedene, forsøk å angi hvor mange oppdrag du har måttet bruke tvang for at en pasient skulle være forsvarlig sikret under transport?

*Dette elementet vises kun dersom alternativet «Ja» er valgt i spørsmålet «Jobber du i operativ tjeneste på ambulanse som utfører akutt- og transportoppdrag?»*

For eksempel ved å: ta på pasienten sikkerhetsbeltene hvis pasienten forsøker å komme løs av dem holde pasientens armer og/eller ben fast uten pasientens samtykke

0
1
2
3
4
5
6
7
8
9
10 eller flere

I løpet av de siste seks månedene, forsøk å angi hvor mange oppdrag du har festet/sikret pasientens armer og/eller ben ved hjelp av tepper/bandasjer/borrelåsstropper etc for at vedkommende skulle være forsvarlig sikret under ambulansetransport?

*Dette elementet vises kun dersom alternativet «Ja» er valgt i spørsmålet «Jobber du i operativ tjeneste på ambulanse som utfører akutt- og transportoppdrag?»*

0
1
2
3
4
5
6
7
8
9
10 eller flere

I løpet av de siste seks månedene, forsøk å angi hvor mange oppdrag du har opplevd at ambulansepersonellet/politiet/andre har sittet uten sikkerhetsbelte for å holde en pasient i ro under ambulansetransport?

*Dette elementet vises kun dersom alternativet «Ja» er valgt i spørsmålet «Jobber du i operativ tjeneste på ambulanse som utfører akutt- og transportoppdrag?»*

0
1
2
3
4
5
6
7
8
9
10 eller flere

**Videre er spørsmålene IKKE avgrenset til en spesifikk tidsperiode.**

Har du noen gang i løpet av din yrkeskarriere festet/sikret pasientens armer og/eller ben ved hjelp av tepper/bandasjer etc for at vedkommende skulle være forsvarlig sikret under ambulansetransport?

*Dette elementet vises kun dersom alternativet «Ja» er valgt i spørsmålet «Jobber du i operativ tjeneste på ambulanse som utfører akutt- og transportoppdrag?»*

Ja
Nei
Vet ikke

Har du noen gang i løpet av din yrkeskarriere brukt tvang på andre måter enn beskrevet i tidligere spørsmål for å sikre pasienter under ambulansetransport?

*Dette elementet vises kun dersom alternativet «Ja» er valgt i spørsmålet «Jobber du i operativ tjeneste på ambulanse som utfører akutt- og transportoppdrag?»*

Hvis ja, fortell med egne ord [fritekst] (Vennligst ikke oppgi informasjon som kan identifisere deg eller øvrige enkeltpersoner, verken direkte eller indirekte)

Hvordan opplever du det, når det må brukes tvang for å få sikret en pasient under ambulansetransport?

*Dette elementet vises kun dersom alternativet «Ja» er valgt i spørsmålet «Jobber du i operativ tjeneste på ambulanse som utfører akutt- og transportoppdrag?»*

Angi din opplevelse av slike transporter fra helt uproblematisk til svært ubehagelig på den linære skalaen [bilde av nummerisk skala fra 0-10]

Kan du si noe om hvordan du opplever transporter av pasienter hvor det må brukes tvang for å få sikret pasienten under transporten?

*Dette elementet vises kun dersom alternativet «Ja» er valgt i spørsmålet «Jobber du i operativ tjeneste på ambulanse som utfører akutt- og transportoppdrag?»*

Fortell med egne ord [fritekst] (Vennligst ikke oppgi informasjon som kan identifisere deg eller øvrige enkeltpersoner, verken direkte eller indirekte)
