# Supplementary material for: Ambulance personnel use of coercion and use of safety belts in Norway
Source: BMC Health Serv Res. 2023 Nov 27;23:1303. doi: 10.1186/s12913-023-10332-x (PMC10680207; doi:10.1186/s12913-023-10332-x)
Supplement: Supplementary file 3 — Additional file 3. [file 12913_2023_10332_MOESM3_ESM.docx]

**Supplementary file:** Coding of the variables:

**Number of callouts where escorts were not seated with their safety belts fastened** coded as 0 to >10**.**

**Participant age** coded as <19 years = 1; 20–24 years = 2; 25–29 years = 3; 30–34 years = 4; 35–39 years = 5; 40–44 years = 6; 45–49 years = 7; 50–54 years = 8; 55–59 years = 9; 60-64 years = 10; and >64 years = 11.

**Participant gender**, coded as male = 1, female = 0.

**Education level**, coded as lower/upper secondary school, usually students in an apprenticeship = 1; emergency medical technician = 2; up to two years of university level education = 3; up to four years of university level education = 4; four years or more of university education = 5.

**Years of experience** from ambulance services was collected in groups of years to ensure anonymity and coded as <1 years = 0; 1–2 years = 2; 3–4 years = 4; 5–6 years = 6; 7–8 years = 8; 9–10 years = 10; 11–12 years = 12; 13–14 years = 14; 15–16 years = 16; 17–18 years = 18; 19–20 years = 20; >20 years = 22.

**Working hours** is measured as “close to full-time position” = 25,5–33,5 hours per week (coded as 4); “51–75% position” = 17–25 hours per week (coded as 3); “26–50% position” = 9–17 hours per week (coded as 2); “< 26% position” = <17 hours per week (coded as 1).

**Experience of use of coercion**. coded as 0 = completely unproblematic and 10 = very uncomfortable.
